# Impact of *Streptococcus salivarius* K12 probiotic on pH and tooth enamel integrity in an *in vitro* oral biofilm system

**DOI:** 10.3389/froh.2026.1893799

**Published:** 2026-09-23

**Authors:** Livia Göldi, Viktoriya Shyp, Klaus W. Neuhaus, Markus Reise, Michael M. Bornstein, Elisabeth Reichardt

**Affiliations:** 1 Department Research, University Center for Dental Medicine UZB, University of Basel, Basel, Switzerland; 2 Department of Restorative, Preventive and Pediatric Dentistry, School of Dental Medicine, University of Bern, Bern, Switzerland; 3 Department of Conservative Dentistry and Periodontology, Center of Dental Medicine, Jena University Hospitals, Jena, Germany; 4 Department of Oral Health & Medicine, University Center for Dental Medicine UZB, University of Basel, Basel, Switzerland; 5 Section of Orthodontics, Department of Odontology, Faculty of Health and Medical Sciences, University of Copenhagen, Copenhagen, Denmark; 6 Department of Pediatric Oral Health and Orthodontics, University Center for Dental Medicine UZB, University of Basel, Basel, Switzerland

**Keywords:** biofilm, bovine enamel, demineralization, extracellular matrix, pH, probiotics

## Abstract

**Aim:**

To evaluate the impact of an early exposure and a single re-administration of the probiotic *Streptococcus salivarius* K12 on pH dynamics, bacterial viability, and enamel surface hardness in a multispecies cariogenic biofilm over 12 days under the sucrose challenging conditions.

**Methods:**

Anaerobic co-cultures of five oral streptococcal species (*S. mutans*, *S. sobrinus*, *S. sanguinis*, *S. mitis*, and *S. gordonii*) were grown with or without *S. salivarius* K12 on bovine enamel specimens in artificial saliva (AS) in well plates at 37 °C. The static biofilm model was exposed daily to AS with 0.5% sucrose for 8 hours, followed by AS without sucrose for 16 hours. *S. salivarius* K12 was applied at initial inoculation and re-administered on day 3. Planktonic pH and colony-forming units (CFU/mL) were measured before each medium change. On day 12, enamel Martens hardness (HM), biofilm cell viability (CFU/mL), and extracellular matrix pH (C-SNARF-4 ratiometry) were assessed. Probiotic-only and AS-only conditions served as controls.

**Results:**

A single reapplication of *S. salivarius* K12 to the mixed cariogenic culture significantly reduced acidification by day 3–4 with pH values 5.9 ± 0.26 compared to 4.4 ± 0.06 in culture without probiotic. However, this effect diminished over time. By day 12, no significant differences in HM, extracellular biofilm pH, or CFU/mL were observed between probiotic-treated and untreated cultures.

**Conclusions:**

Early exposure and a single re-administration of *S. salivarius* K12 can temporarily reduce acidification in artificial cariogenic biofilms, however repeated applications is necessary for lasting effects on enamel integrity and biofilm properties.

## 1 Introduction

Dental caries is a biofilm-mediated, diet-modulated disease that arises from ecological shifts within the dental biofilm under conditions of frequent exposure to fermentable carbohydrates (1, 2). Repeated sugar intake promotes sustained acidification at the tooth surface, favoring the selection of acidogenic and aciduric microorganisms and driving enamel demineralization (3–6). Within this ecological framework, contemporary preventive strategies increasingly aim not only to reduce microbial load but to modulate biofilm composition and function toward a health-associated state (7).

Conventional adjunctive approaches to biofilm control, including broad-spectrum antimicrobials such as chlorhexidine, exhibit limited specificity and may not support the re-establishment of a balanced oral microbiome. Their prolonged use has also been associated with undesirable side effects and potential contributions to antimicrobial resistance (8, 9). These limitations have prompted growing interest in ecological approaches, including the use of probiotics, to selectively influence oral biofilms without disrupting beneficial microbial communities (10–12).

Among candidate oral probiotics, *Streptococcus salivarius* K12 has attracted attention due to its natural occurrence in the oral microbiome and its capacity to produce bacteriocin-like inhibitory substances (BLIS), which can suppress susceptible oral streptococci (12–14). In addition to antimicrobial activity, *S. salivarius* has been suggested to contribute to oral pH homeostasis through alkali-generating pathways and ecological competition (15–18). However, the functional relevance of these properties under cariogenic conditions remains uncertain, as *S. salivarius* itself may be sensitive to acidic environments and may exhibit reduced activity under sustained low pH conditions (19).

Despite increasing interest in probiotics for oral health, current evidence in cariology remains heterogeneous. Clinical studies have reported variable effects on microbial markers, often based on short-term observations or surrogate endpoints (18). *In vitro* investigations frequently rely on simplified mono- or dual-species systems that do not capture the structural and metabolic complexity of mature dental biofilms (19). Consequently, it remains unclear whether probiotic interventions can meaningfully influence mature multispecies cariogenic biofilms, particularly under repeated sucrose challenge and in relation to clinically relevant outcomes such as enamel demineralization. In our previous *in vitro* study using a oral flow chamber model, *S. salivarius* K12 inhibited *S. mutans* viability and biofilm formation in a simplified dual-species system over a short-term period (20). Similar studies have primarily focused on short-term antimicrobial effects or simplified biofilm models, leaving the long-term functional performance of probiotics in maturing cariogenic biofilms largely unexplored. It therefore remains unclear whether these early effects translate into sustained modulation of biofilm acidogenicity, bacterial viability, and enamel demineralization during prolonged biofilm maturation under continuous cariogenic challenge (21, 22).

To address this, the present study used a static multispecies biofilm model in well plates, incorporating periodic medium replacement and repeated sucrose exposure using pipette tips on bovine enamel specimens to mimic frequent dietary challenges. A 12-day experimental period was selected to enable biofilm maturation, including the establishment of acidogenic and aciduric communities, extracellular matrix accumulation, and stable pH gradients characteristic of cariogenic plaque (23). These features are not achieved in short-term models but are essential for assessing whether early microbial interventions exert lasting effects.

The aim of this study was to investigate whether early-stage exposure to *S. salivarius* K12 affects the development of mature cariogenic multispecies biofilms and associated enamel demineralization under prolonged sucrose challenge.

## 2 Materials and methods

### 2.1 Microbial strains and growth conditions

*S. mutans* ATCC 25175, *S. gordonii* ATCC 10558, *S. sanguinis* DSM 20068, *S. sobrinus* ATCC 33402, *S. mitis* ATCC 49456, *S. salivarius* K12 (Burgerstein Biotics-O, Switzerland), *Bacteroides fragilis* ATCC 25285, and *Micrococcus luteus* strains (6575, 6592) were used. Strains were stored at −80°C and cultured on Columbia blood agar supplemented with 5% human blood (Blood donor center, Basel, Switzerland) under anaerobic conditions (80% N₂, 10% H₂, 10% CO₂) at 37°C for 24 h.

### 2.2 Biofilm formation on bovine enamel

Seventy-two caries-free bovine incisors were used in this study. For each methodological analysis (CFU/pH, SEM, and enamel hardness), 24 enamel specimens were allocated to four experimental groups (6 specimens per group). These specimens were distributed across three independent biological experiments, each including two enamel specimens per group. Each biological experiment was performed independently using a fresh bacterial inoculum and a new set of enamel specimens. The two specimens within each group were averaged to generate one biological data point. Statistical analyses were therefore based on three independent biological replicates (*n* = 3).

Specimens were prepared, embedded in resin, and polished with Siliciumcarbid (SiC) discs of grain size P800, P1200 and P2400 (Presi, Eybens, France) 90 s each to remove ∼200 µm enamel. Specimens were stored in mineral solution (1.5 mmol/L CaCl2, 1.0 mmol/L KH2PO4, 50 mmol/L NaCl, pH = 7.0) and sonicated prior to use. Samples were randomly assigned to four groups: (1) control (artificial saliva, AS), (2), *S. salivarius* K12, (3) streptococci + K12, and (4) streptococci (24).

A static *in vitro* biofilm model was established in 24-well plates (Thermo Fisher, Waltham, MA, USA), where medium was periodically replaced using pipette tips to evaluate the pH-modulating and antimicrobial effects of *S. salivarius* K12 on a five-species oral streptococcal biofilm, with and without sucrose exposure (Figure 1) (25). Single colonies grown on Columbia blood agar were transferred to 15 mL artificial saliva (AS) containing 0.5% sucrose and incubated anaerobically at 37°C for 12 h (26). Bacterial suspensions were adjusted to 1.00E + 07 CFU/mL, resuspended in 0.9% NaCl, ultrasonicated (30 s, 22.5 W), centrifuged (9200 rpm, 5 min), and pellets were resuspended in AS with 0.05% sucrose. For co-cultivation, *S. mutans*, *S. gordonii*, *S. sanguinis*, *S. sobrinus*, and *S. mitis* were mixed either with *S. salivarius* K12 (group 3; 1:1:1:1:1:1) or without (group 4; 1:1:1:1:1). Group 2 contained *S. salivarius* K12 only, and group 1 (AS without bacteria) served as control (Figure 1). Enamel specimens were placed in 24-well plates, and biofilm formation was initiated with 1 mL AS containing 0.5% sucrose with or without bacteria (groups 1–4). After 24 h (T0), the medium was carefully replaced daily using pipette tips with AS containing 0.5% sucrose for 8 h and AS without sucrose for 16 h under anaerobic conditions (Figures 1, 2). To evaluate long-term effects, the model was maintained for 12 days. On day 3, *S. salivarius* K12 (1.00E + 07 CFU/mL) was reintroduced for 8 h in groups 2 and 3. At day 12 (T1), planktonic and biofilm-associated bacteria were collected. Biofilms were harvested by swabbing, resuspended in AS, vortexed, and plated for CFU determination. All experiments were performed in biological triplicates.

**Figure 1:**
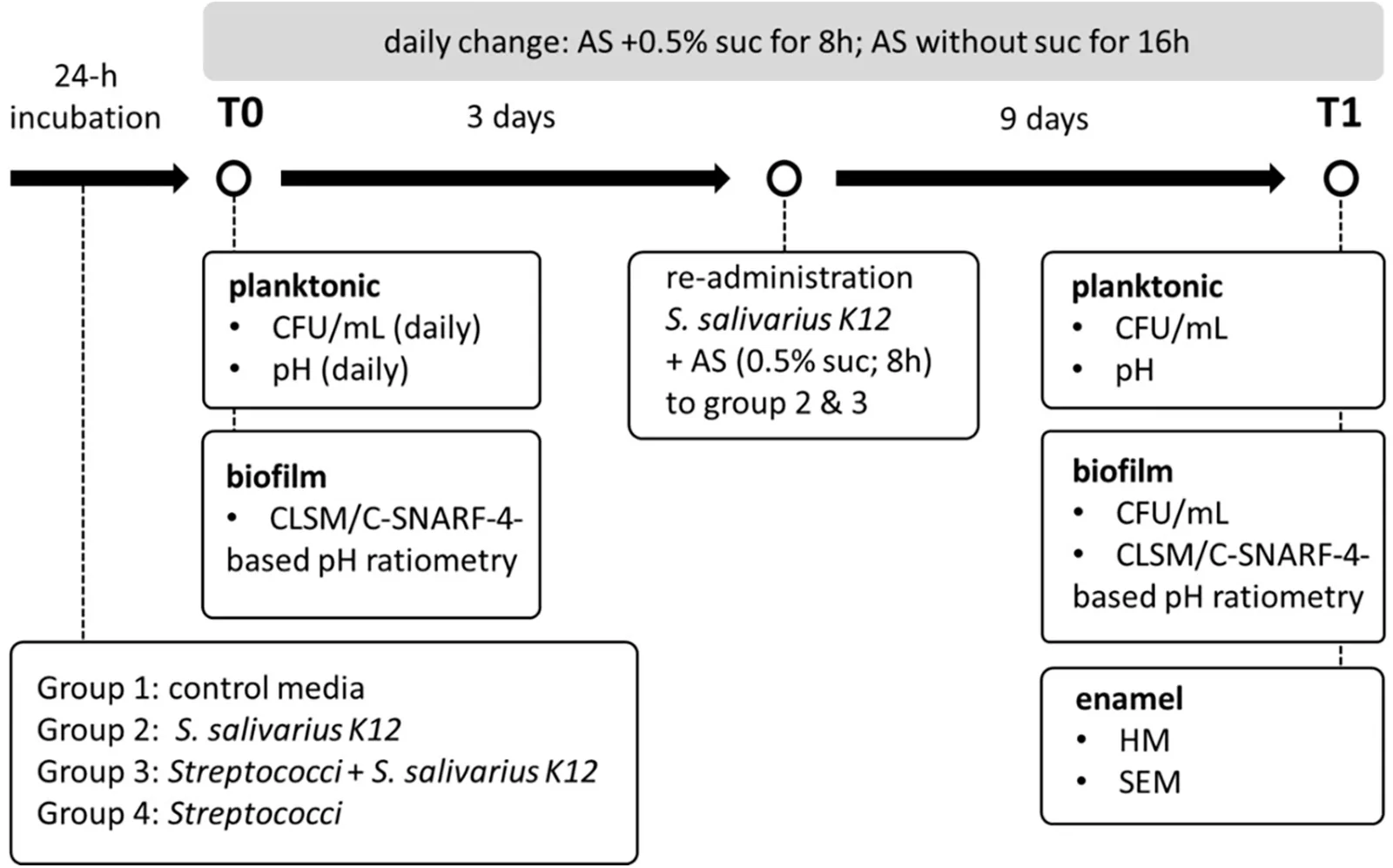
Experimental scheme of the sucrose-challenge model.

**Figure 2:**
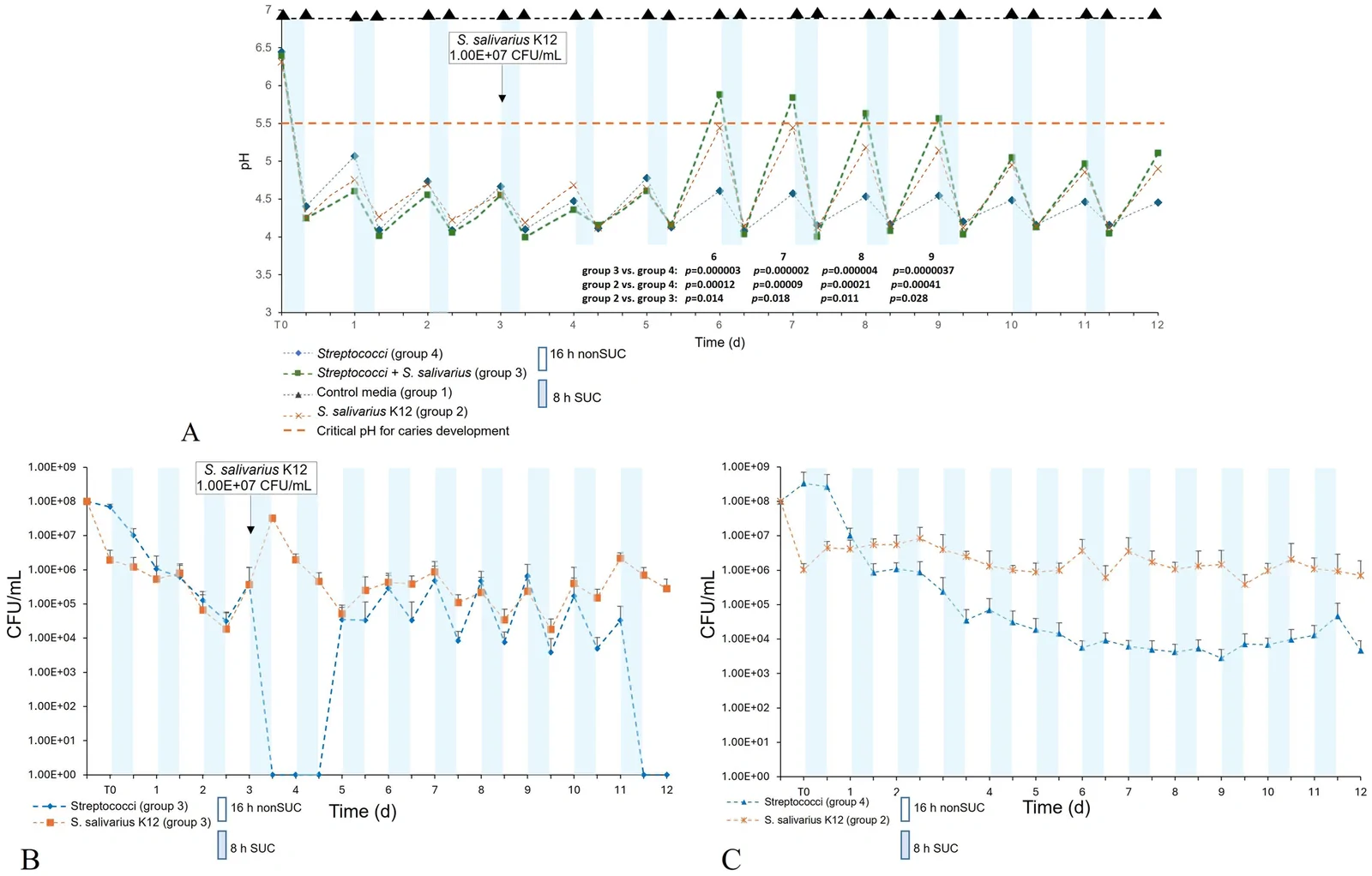
Temporal changes in pH **(A)** and planktonic CFU counts **(B–C)** during bacterial co-cultivation under sucrose-challenging conditions.

### 2.3 pH measurements (planktonic and biofilm)

The pH of planktonic suspensions was measured daily after 8 h incubation in artificial saliva (AS) supplemented with 0.5% sucrose and after 16 h incubation in AS without sucrose using a pH electrode (Figure 1). For modified pH ratiometry, biofilms were formed in 100 µL artificial saliva (AS) in glass-bottom 96-well plates under the sucrose-challenging conditions described above and incubated anaerobically at 37°C. At T0 (24 h) and T1 (12 days), C-SNARF-4 dye (20 µM) was added for pH measurement. For calibration, MES buffer solutions (50 mM) were adjusted to pH 4–8 (0.2 increments) and mixed with C-SNARF-4 (20 µM) (27). Images were acquired at six predefined fields of view (1000 µm apart, 5 µm above the biofilm bottom) using a 63×/1.40 oil immersion objective (Zeiss, Germany). This experiment was performed in three independent biological replicates (*n* = 3). Each biological replicate consisted of two technical replicates, which were averaged to generate one data point for statistical analysis.

### 2.4 Enumeration of viable bacteria using colony-forming units

Bacteria were quantified by serial tenfold dilution in 0.9% NaCl and plating of 100 µL aliquots on Mitis Salivarius Agar (MSA) (Difco™ Mitis Salivarius Agar, BD, Allschwil, Switzerland) for selective enumeration of streptococci. This morphological differentiation allowed reliable discrimination of *S. salivarius* K12 (big colonies) from other streptococci (small colonies) present in the samples (Supplementary Figure 1) (20, 28). CFU/mL were determined at T0 and twice daily after 8 h (with 0.5% sucrose) and 16 h (without sucrose) incubation. Counts were obtained for *S. salivarius* K12 and total streptococci.

### 2.5 Antimicrobial activity of *S. salivarius* K12: simultaneous antagonism method

The antimicrobial activity of *S. salivarius* K12 is a key indicator of its bacteriocin (BLIS) production and was assessed using the simultaneous antagonism method due to previously reported BLIS-mediated antagonism against indicator strains *M. luteus* (6575, 6592) and the negative control *B. fragilis* (17, 20, 29). Indicator strains were grown overnight in Todd-Hewitt Broth and adjusted to McFarland 0.5. Aliquots (100 µL) were spread on CaCO₃ (CBA) plates, and *S. salivarius* K12 colonies were stabbed into the agar. Plates were incubated anaerobically at 37°C for 48 h. Zones of inhibition were measured and classified as: none (0 mm, “−”), slight (1–2.5 mm, “+”), moderate (2.5–4 mm, “++”), or strong (4–11 mm, “+++”) (Table 2) (20).

### 2.6 Scanning electron microscopy (SEM)

After 12 days (T1), enamel specimens were analyzed by SEM. Attached bacteria were removed by vortexing in 0.9% NaCl for 1 min. Specimens were fixed overnight in 2% glutaraldehyde, washed with PBS, and dehydrated in graded ethanol (30%–100%, 10 min each). Samples were critical-point dried, gold-coated (10 nm), and examined by SEM (Fei Nova NanoSEM 230®) (20). Three random areas per specimen were imaged at 200×, 2000×, and 5000× magnification.

### 2.7 Martens hardness of enamel surfaces

Martens hardness (HM) of enamel surfaces was measured at T1 after biofilm removal using a hardness-testing machine (Z2.5, ZwickRoell, Germany). Measurements were performed on planar specimens with a Vickers indenter under a load of 19.62 N (loading speed 5 N/s, dwell time 2 s, unloading speed 30 mm/min). Force-indentation depth curves were recorded to determine material parameters. HM (N/mm²) was calculated as the applied force (F) divided by the indentation surface area (As), derived from the maximum indentation depth (h) (30). Force, depth, and time were continuously recorded during indentation.

$$HM=\frac{F}{As(h)}$$

Force, depth, and time were continuously recorded during indentation.

### 2.8 Data analysis and statistics

All experiments were performed in independent biological triplicates. Normality of residuals was confirmed using the Shapiro–Wilk test, and homogeneity of variance using Levene’s test. Data were analyzed using one- or two-way ANOVA with Tukey’s *post hoc* test, calculated and visualized in GraphPad Prism (San Diego, California, USA). Exact *p*-values, 95% confidence intervals, and effect sizes (*η*²) are reported where applicable. Differences were considered significant at *p*< 0.05 (*), *p*< 0.01 (**), *p*< 0.001 (***), and *p*< 0.0001 (****).

## 3 Results

### 3.1 Transient effects of *S. salivarius* K12 on acidification in the sucrose-challenging model

The sucrose-challenging model induced pronounced cyclic fluctuations in planktonic pH across all experimental groups (Figure 2A). During the initial 8 h exposure to artificial saliva (AS) supplemented with 0.5% sucrose, pH decreased from 6.4 ± 0.99 [CI: 3.94, 8.86] at baseline (T0) to 4.29 ± 0.09 [CI: 4.07, 4.51]. During the subsequent 16 h incubation in sucrose-free AS, partial pH recovery occurred; however, values remained below the critical threshold of 5.5. This pattern of acidification and incomplete recovery persisted until day 6 in all groups. Following re-administration of *S. salivarius* K12 to group 3 on day 3, a delayed and transient increase in pH was observed during the sucrose-free incubation phases between days 6 and 9. During these periods, pH values in group 3 temporarily exceeded the critical threshold of 5.5 (5.89 ± 0.23 [CI: 5.32, 6.46], 5.84 ± 0.29 [CI: 5.12, 6.56], 5.63 ± 0.36 [CI: 4.74, 6.52], and 5.56 ± 0.36 [CI: 4.67, 6.45] on days 6–9, respectively. A similar but less pronounced pattern was observed in group 2. In contrast, the cariogenic streptococcal control (group 4) consistently exhibited lower pH values throughout the experiment. From day 9 onward, pH remained below 5.5 in all groups and did not recover to non-cariogenic levels (Figure 2A).

At 24 h after initial inoculation (T0), extracellular matrix pH values had decreased in all bacterial groups (Figure 3A; Table 1). pH ratiometry revealed values of 5.40 ± 0.04 (bottom) and 5.21 ± 0.15 (top) for the *S. salivarius* K12 monoculture (group 2), 5.08 ± 0.15 (bottom) and 5.21 ± 0.08 (top) for the co-cultivation group (group 3), and 5.37 ± 0.45 (bottom) and 5.28 ± 0.42 (top) for the streptococcal control (group 4) with an effect size *η*2 = 0.22 (bottom) and *η*2 = 0.002 (top). After 12 days of cultivation (T1), extracellular matrix pH had further decreased in all groups except group 3, which maintained values close to initial levels (5.24 ± 0.09 bottom; 5.24 ± 0.07 top; effect size *η*2 = 0.010 (bottom) and *η*2 = 0.25 (top) (Figure 3A; Table 1).

**Figure 3:**
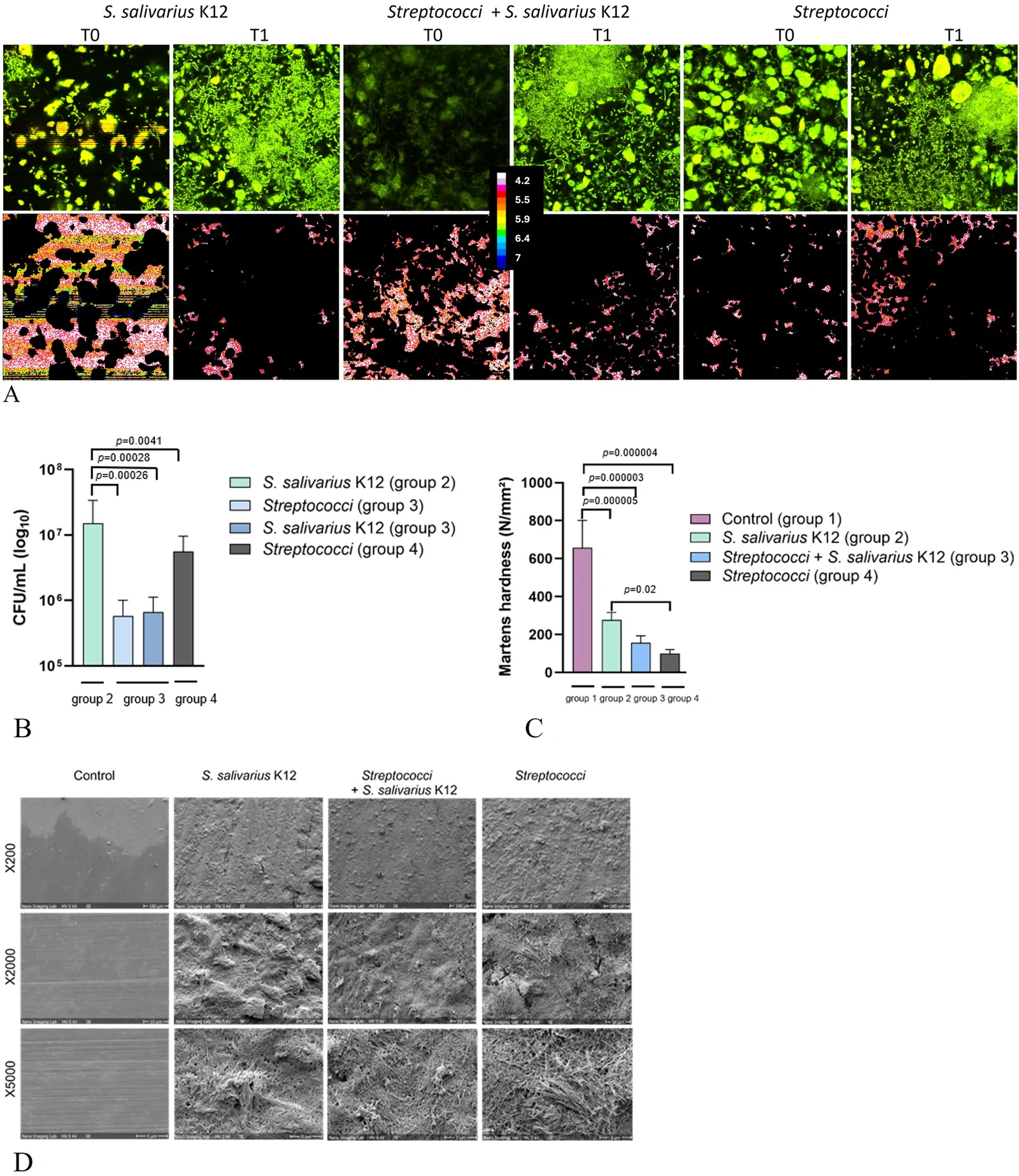
Multimodal analysis of biofilm activity and enamel surface integrity following bacterial co-cultivation.

**Table 1:** ph development over time in biofilms stained with C-SNARF-4. Images were recorded at two time points: after 24 hours of initial inoculation (T0) and on day 12 (T1) in sucrose-challenging model. Data are shown for three groups: *S. salivarius* K12 monoculture (Group 2), mixed streptococci with *S. salivarius* K12 (Group 3), and five-species streptococcal biofilm without *S. salivarius* (Group 4) (*n* = 3).

Time point	*S. salivarius* K12 (group 2) ± Std Dev/[CI]	*p* value (T0-T1)	*Streptococci* + *S. salivarius* K12 (group 3) ± Std Dev/[CI]	*p* value (T0-T1)	*Streptococci* (group 4) ± Std Dev/[CI]	*p* value (T0-T1)
T0 (bottom)	5.40 ± 0.04 [5.30, 5.50]	*p* = 0.38	5.08 ± 0.15 [4.71, 5.45]	*p* = 0.91	5.37 ± 0.45 [4.26, 6.48]	*p* = 0.59
T1 (bottom)	5.06 ± 0.68 [3.37, 6.75]		5.24 ± 0.09 [5.12, 5.36]		5.11 ± 0.05 [4.98, 5.24]	
T0 (top)	5.21 ± 0.15 [4.84, 5.58]	*p* = 0.98	5.21 ± 0.08 [5.01, 5.41]	*p* = 0.94	5.28 ± 0.42 [4.23, 6.33]	*p* = 0.77
T1 (top)	5.22 ± 0.05 [5.09, 5.35]		5.24 ± 0.07 [5.06, 5.42]		5.08 ± 0.05 [4.95, 5.21]	

### 3.2 *S. salivarius* K12 induced limited and time-dependent shifts in bacterial viability

During the first 3 days of incubation, planktonic CFU counts decreased to varying extents in all bacterial groups (Figures 2B,C). Administration of *S. salivarius* K12 (1.00E + 07 CFU/mL) to group 2 on day 3 did not significantly alter planktonic CFU counts thereafter (Figure 2C). In contrast, re-administration of the probiotic to cariogenic streptococci in group 3 was associated with a short-term reduction in planktonic CFU counts of the cariogenic strains within the subsequent 24 h, while CFU values for *S. salivarius* K12 remained elevated (Figure 2B). From day 5 onward, CFU values of *S. salivarius* K12 and cariogenic streptococci were largely comparable between groups until day 11, when differences re-emerged (Figure 2B).

To assess viability within mature biofilms formed on bovine enamel specimens, CFU counts of *S. salivarius* K12 and cariogenic streptococci were determined after 12 days of cultivation (Figure 3B). At T1, biofilm CFU/mL values in the co-cultivation group (group 3) were comparable between *S. salivarius* K12 and cariogenic streptococci. In contrast, higher CFU/mL values were observed in the respective monoculture groups (groups 2 and 4).

### 3.3 Antimicrobial activity of *S. salivarius* K12 - simultaneous antagonism method

Using a simultaneous antagonism assay, *S. salivarius* K12 exhibited antimicrobial activity against all strains used in this study (Table 2). In addition, it effectively inhibited indicator strains *M. luteus* 6575, *M. luteus* 6592.

**Table 2:** Inhibition of bacterial growth by *S. salivarius* K12 as determined by the simultaneous antagonism test.

Indicator strains	*S. salivarius* producer strain K12
*S. mutans* ATCC 25175	++
*S. sobrinus* ATCC 33402	+
*S. gordonii* ATCC 10558	+
*S. sanguinis* DSM 20068	+
*S. mitis* ATCC 49456	+
*M. luteus* 6575	+++
*M. luteus* 6592	+++
*S. salivarius* K12	−
*B. fragilis*	−

“−”- No inhibition; “+” - Slight inhibition (1–2.5 mm); “++” - Inhibition (2.5–4 mm); and “+++” - Strong inhibition (4–11.0 mm).

### 3.4 Effects of early probiotic exposure on enamel hardness loss

Descriptive scanning electron microscopy (SEM) evaluation revealed enamel surface alterations in all bacterial groups after 12 days compared with the artificial saliva control (group 1) (Figure 3D). Biofilm-exposed specimens showed features consistent with demineralization, including surface irregularities and partial prism exposure. These changes appeared more pronounced in the cariogenic streptococcal group (group 4), while groups 2 and 3 showed similar but less extensive alterations. The control group displayed a smooth enamel surface.

Enamel hardness measurements (HM) confirmed significant demineralization in all bacterial groups compared with the control (*p* = 0.000005; *p* = 0.0000039; *p* = 0.000004) (Figure 3C). The greatest reduction was observed in group 4. Importantly, the addition of *S. salivarius* K12 (group 3) did not significantly reduce hardness loss compared with the cariogenic control (*p* > 0.05). Group 2 showed a smaller reduction than group 4 (*p* = 0.02), but values remained significantly lower than in the control. No significant difference was observed between groups 2 and 3.

## 4 Discussion

The present study investigated whether early exposure to *S. salivarius* K12 can influence the development of mature multispecies cariogenic biofilms and associated enamel changes under repeated sucrose exposure. By extending previous short-term investigations in simplified biofilm systems to a prolonged multispecies cariogenic model, this study provides new insight into the durability of probiotic effects under sustained cariogenic conditions. The findings indicate that *S. salivarius* K12 exerts transient effects on biofilm acidification changes and planktonic bacterial viability; however, these effects were insufficient to reduce enamel hardness loss in a mature cariogenic biofilm model (Figure 3).

In the applied sucrose-challenging system, all bacterial groups exhibited pronounced acidogenicity, including the *S. salivarius* K12 monoculture (Figure 3A). Although *S. salivarius* is generally considered less acidogenic than classical cariogenic streptococci, the present data indicate that under high sucrose conditions it still contributes to environmental acidification (31–33). This observation aligns with the ecological plaque hypothesis, which proposes that frequent carbohydrate exposure drives environmental acidification and a shift in the microbiota, whereby even health-associated species can contribute to acid production and support a more aciduric community (2).

Within this context, *S. salivarius* K12 did not prevent the establishment of an acidic biofilm environment but transiently attenuated acidification, primarily during sucrose-free phases (Figure 2A). The transient increase in pH is likely multifactorial. During sucrose-free periods, reduced acid production temporarily allowed the lower acidogenicity and alkali-generating metabolism of *S. salivarius* K12 to shift the local pH toward neutrality. Following renewed sucrose exposure, however, highly acidogenic and aciduric species rapidly re-established a low-pH environment. This interpretation is consistent with the ecological plaque hypothesis, whereby repeated sugar exposure continually selects for acidogenic metabolism and limits the persistence of health-associated ecological functions. Accordingly, the pH-modulating capacity of *S. salivarius* K12 appears to be highly context-dependent and insufficient to counteract sustained cariogenic pressure (34).

The effects on bacterial viability further support a short-lived probiotic impact. A transient reduction in planktonic cariogenic streptococci was observed following probiotic administration, but this effect diminished within 24-48 hours, and no substantial differences in CFU counts were detected in mature biofilms after 12 days (Figures 2A, B). This pattern likely reflects an initial suppression driven by *S. salivarius* K12 through BLIS-mediated antimicrobial activity and short-term microenvironmental shifts, followed by rapid streptococcal regrowth once inhibitory pressure declined, underscoring the resilience of oral streptococci and the need for repeated probiotic exposure (Figure 2B) (20). First, the prolonged acidic environment generated by repeated sucrose exposure may have compromised the survival and metabolic activity of *S. salivarius* K12, thereby reducing its competitiveness against highly aciduric species such as *S. mutans* and *S. sobrinus* (33). Second, successful probiotic activity depends on stable colonization and persistence within the developing biofilm. Under repeated sucrose exposure, strong biofilm-forming species such as *S. mutans* and *S. sobrinus* are likely to outcompete *S. salivarius* K12 for binding sites and nutrients, limiting its establishment within the maturing biofilm and consequently its sustained probiotic activity. Finally, the antagonistic activity of *S. salivarius* K12 relies on salivaricin and other antimicrobial compounds, and because all bacteriocin-producing S. salivarius strains carry large mobile plasmids encoding multiple lantibiotic loci, these plasmids likely act as transferable repositories whose expression may be regulated by environmental conditions (35).

Collectively, these mechanisms may explain why antagonistic effects observed in simplified *in vitro* assays did not translate into persistent suppression of cariogenic streptococci within a more complex, maturing biofilm over the 12-day study period. However, further studies incorporating analyses of biofilm composition, extracellular matrix production, and bacterial gene expression are needed to determine whether *S. salivarius* K12 exerts longer-term ecological effects on the developing biofilm (16, 22).

Consistent with the observed acidification patterns, all bacterial groups induced significant enamel hardness loss. Importantly, the addition of *S. salivarius* K12 to the cariogenic consortium did not reduce enamel hardness loss compared with the cariogenic control (Figure 3D). While SEM imaging suggested minor qualitative differences, these changes were not corroborated by quantitative hardness measurements (Figure 3C). Together, these findings indicate that the observed transient modulation of biofilm medium acidification and planktonic viability did not translate into measurable protection against enamel demineralization under sustained cariogenic conditions (Figure 3). From an ecological and translational perspective, the limited efficacy of *S. salivarius* K12 as a single early intervention underscores the challenge of reshaping an environment that remains strongly cariogenic. Probiotic strains must not only inhibit key pathogens but also remain metabolically active and competitive under low pH and high sugar availability. In its current form, *S. salivarius* K12 appears insufficiently robust under these conditions. Thus, the present findings support the concept that probiotic strategies will likely be most effective when integrated into multimodal ecological approaches rather than used alone. Combining oral probiotics with agents that promote biofilm alkalization, such as arginine-containing formulations, could raise environmental pH and improve the competitiveness of health-associated species (19, 26). In parallel, regular mechanical biofilm disruption through tooth brushing and interdental cleaning reduces biomass and ecological niches for *S. mutans* and related pathogens, potentially creating a more receptive environment for probiotic colonization and activity. In future, tailored combinations of probiotics, pH-modulating formulations, and conventional mechanical plaque control may offer a more powerful means of maintaining a health-associated biofilm in high-risk individuals.

Several limitations of this investigation should be acknowledged. Although the static multispecies biofilm model in well plates, incorporating periodic medium replacement, was designed to simulate oral conditions, it cannot fully reproduce host related factors such as saliva flow, immune responses, or mechanical disruption of plaque during daily oral hygiene. The microbial community was restricted to selected streptococcal species, and broader ecological interactions were not assessed. The limited experimental study period of 12 days captures biofilm maturation and early enamel changes under cariogenic challenge but does not allow conclusions regarding long term probiotic performance, adaptation, or community resilience (26). Furthermore, *S. salivarius* K12 was administered only during the early stages of biofilm development, and the present study was not designed to compare different dosing regimens. Consequently, no conclusions can be drawn regarding the optimal frequency or duration of probiotic administration. Additionally, biofilms were not mechanically disturbed, as would occur during routine brushing. Future studies should therefore incorporate more complex microbial consortia, extended observation periods, systematic comparisons of probiotic dosing schedules (including repeated or daily administration), and rationally designed combination therapies to more fully explore the time- and context-dependent potential of probiotics in caries prevention.

## 5 Conclusion

Early exposure of cariogenic biofilm to *S. salivarius* K12 induced transient modulation of biofilm acidification and bacterial viability but did not prevent enamel demineralization under sustained cariogenic conditions, indicating limited efficacy as a single intervention in mature multispecies biofilms.

## Data Availability

The raw data supporting the conclusions of this article will be made available by the authors, without undue reservation.
