# Supplementary material for: Impact of Streptococcus salivarius K12 probiotic on pH and tooth enamel integrity in an in vitro oral biofilm system
Source: Front Oral Health. 2026 Sep 23;7:1893799. doi: 10.3389/froh.2026.1893799 (PMC13642219; doi:10.3389/froh.2026.1893799)
Supplement: Supplementary file 1 [file Supplementaryfile1.docx]

Supplementary Material

# 1 Supplementary Figures

**Figure 1**. Example of analyzing *S. salivarius* K12 (group 3, big colonies) colony forming units (CFU/mL) within co-cultivation of all *streptococci* (group 3, small colonies) after 48h using Mitis Salivarius Agar (MSA, Difco™ Mitis Salivarius Agar, BD, Allschwil, Switzerland) under anaerobic conditions. Colony forming units were quantified by serial tenfold dilution in 0.9% NaCl and plating of 100 µL aliquots for selective enumeration of *streptococci*.
